# Evaluation of Rectal Varices by Endoscopic Ultrasonography in Patients With Portal Hypertension

**DOI:** 10.1155/DTE.7.165

**Published:** 2001

**Authors:** Hiroyuki Kobayashi

**Affiliations:** 1 The 3rd Department of Internal Medicine Toho University School of Medicine Ohashi Hospital 2-17-6, Ohashi, Meguro-Ku Tokyo 153-8515 Japan; 2 Internal Medicine Tokyo Kyousai Hospital 2-3-8, Nakameguro Meguro-ku Tokyo 153-8934 Japan

## Abstract

The usefulness of endoscopic ultrasonography (EUS) in the evaluation of rectal varices (RV) was determined in 50 patients with portal hypertension (PH) and 25 PH-free controls. F_1_ and F_2_ varices and angiectasia were specific for the PH group as evaluated by endoscopy, but there was no difference between the PH and the control groups with respect to the frequency of blue vein. The detection rate of submucosal veins (SMV) with EUS was 88% for the PH group and 68% for the control group. The mean SMV diameter was significantly greater for the PH group than for the control group, and no 2-mm or larger SMV was detected in the control group. Serum albumin and cholinesterase levels were significantly higher for the RV(+) patients with SMV 2mm or more in diameter in the PH group than for the RV(–) patients. The spleen index was also significantly higher for the former group. The frequency of RV was significantly higher for advanced PH than for mild PH. RV(+) was detected in about 30% of endoscopically normal patients in the PH group. The results of this study indicate that EUS is useful in detecting RV and evaluating its pathological condition.


*Diagnostic and Therapeutic Endoscopy*, Vol. 7, pp. 165–174
Reprints available directly from the publisher
Photocopying permitted by license only

© 2001 OPA (Overseas Publishers Association) N.V.
Published by license under
the Harwood Academic Publishers imprint,
part of Gordon and Breach Publishing
a member of the Taylor & Francis Group.
All rights reserved.

### Evaluation of Rectal Varices by Endoscopic Ultrasonography in Patients with Portal Hypertension

HIROYUKI KOBAYASHI*

*The 3rd Department of Internal Medicine, Toho University School of Medicine, Ohashi Hospital, 2-17-6, Ohashi, Meguro-Ku, Tokyo 153-8515, Japan*

*(Received 12 November 2001; In final form 22 March 2002)*

The usefulness of endoscopic ultrasonography (EUS) in the evaluation of rectal varices (RV) was determined in 50 patients with portal hypertension (PH) and 25 PH-free controls. $F_1$ and $F_2$ varices and angiectasia were specific for the PH group as evaluated by endoscopy, but there was no difference between the PH and the control groups with respect to the frequency of blue vein. The detection rate of submucosal veins (SMV) with EUS was 88% for the PH group and 68% for the control group. The mean SMV diameter was significantly greater for the PH group than for the control group, and no 2-mm or larger SMV was detected in the control group. Serum albumin and cholinesterase levels were significantly higher for the RV(+) patients with SMV 2 mm or more in diameter in the PH group than for the RV(−) patients. The spleen index was also significantly higher for the former group. The frequency of RV was significantly higher for advanced PH than for mild PH. RV(+) was detected in about 30% of endoscopically normal patients in the PH group. The results of this study indicate that EUS is useful in detecting RV and evaluating its pathological condition.

*Keywords*: Endoscopic ultrasonography (EUS); Portal hypertension; Rectal varices; Submucosal veins

### INTRODUCTION

It is known that plenty of collateral circulation is formed in and out of the liver during the course of portal hypertension (PH) in liver disorders such as cirrhosis. Various collateral vessels form between the portal system and the systemic circulation according to the severity of PH, commonly producing esophageal and gastric varices in the upper gastrointestinal tract. Gastric congestion peculiar to PH is called portal hypertensive gastropathy. In the lower gastrointestinal tract, on the other hand, PH produces a vascular change in the colonic mucosa called portal hypertensive colopathy [1–3]. It was back in 1954

*Address: Internal Medicine, Tokyo Kyousai Hospital, 2-3-8, Nakameguro, Meguro-ku, Tokyo 153-8934, Japan. Tel.: +81-3-3468-1251/3712-3151. Fax: +81-3-3468-1269

TABLE I Characteristics of 50 patients with portal hypertension and 25 control (July 1996–June 2001)

| Characteristics | Patients | Control |
|---|---|---|
| Cases | 50 | 25 |
| Mean age | 65.1 (37–81) | 66.5 (46–88) |
| Sex: M/F | 35/15 | 15/10 |
| Liver disease | | |
| LC | 46 | |
| HCV | 36 | |
| HBV | 1 | |
| Alcohol | 6 | |
| NBNC | 3 | |
| IPH | 3 | |
| EHO | 1 | |
| Child's classification (LC) | | |
| A | 15 | |
| B | 19 | |
| C | 12 | |

LC: liver cirrhosis; IPH: idiopathic portal hypertension; EHO: extrahepatic portal vein obstruction.

that colo-rectal varices were described for the first time [4]. This disease has since been considered important as one of the causes of bleeding from the lower gastrointestinal tract [5]. Rectal varices (RV), among others, once ruptured, usually present great difficulty in diagnosis and treatment, though they occur less often than esophageal varices (EV). Since RV are enlarged veins that are located near the anus and are visualized by endoscopy as a characteristic blue-tinted submucosal tumor-like elevation, diagnosis is relatively easy, but their changes with time still remain obscure and are difficult to evaluate objectively. The literature is sparse on endoscopic ultrasonography (EUS) studies evaluating colonic submucosal veins that are hard to examine by endoscopy. Information available in this area of inquiry has been provided by Dhiman *et al.* [6,7] who studied rectal PH by EUS, Malde *et al.* [8] who investigated RV by transvaginal EUS in PH patients, and Zeniya *et al.* [9,10] who followed RV by EUS. A dedicated ultrasonograph was used in all these studies. In the present study, rectal submucosal veins (SMV) were examined in great detail in PH and non-PH patients using an ultrasound probe to determine whether rectal EUS findings are correlated with endoscopic findings in the reflection of the pathological condition of RV and assess the clinical usefulness of EUS in the evaluation of RV.

### MATERIALS AND METHODS

Fifty patients who were clinically diagnosed as having PH by colonoscopy (combined with EUS) in the period of 5 years from July 1996 to June 2001 were admitted to the study. The patients averaged in the age group 65.1 years (range 37–81 years), consisting of 35 males and 15 females. The cause of PH was liver cirrhosis for 46 patients (HCV 36, HBV 1, alcoholic cirrhosis 6, non-B, non-C hepatitis 3), idiopathic portal hypertension for three patients, and extraheptic portal obstruction for one patient. According to Child-Turcotte's classification of liver cirrhosis, 15 patients had cirrhosis A, 19 cirrhosis B, and 12 cirrhosis C. Besides these patients, 25 patients with no distal lesion to the sigmoid colon were selected from among patients who were examined by colonoscopy for polyps and cancer during the same period of time. These 25 patients served as controls. They averaged 66.5 years and consisted of 15 males and 10 females. There was no bias in age or sex distribution between the PH and the control groups (Table I). The PH group included eight patients with a past history of abdominal surgery and the control group had one similar patient. Prior to entry in the study informed, consent was obtained from all patients.

The apparatus used in this study were colonoscopes (CF-200I, CF-230I, CF-Q240I), ultrasonic endoscopes (CF-UMQ230: 7.5/12 MHz), ultrasonic probes (UM-2R: 12 MHz, UM-3R: 20 MHz, UM-S30-25R: 30 MHz, UM-3D2R: 12 MHz, UM-3D3R: 20 MHz) of Olympus Optical Co., Ltd. In addition, recording unit EU-M30 and image processing unit EU-IP2 were used. EUS was basically conducted with an ultrasound probe, and dedicated apparatus were used for the examination of large or extramural vessels.

The patients were prepared for the colonoscopic examination with oral gastrointestinal cleansing fluids or glycerine enemas. No premedication was used.

Following endoscopic examination in a supine or left recumbent position, EUS was performed by

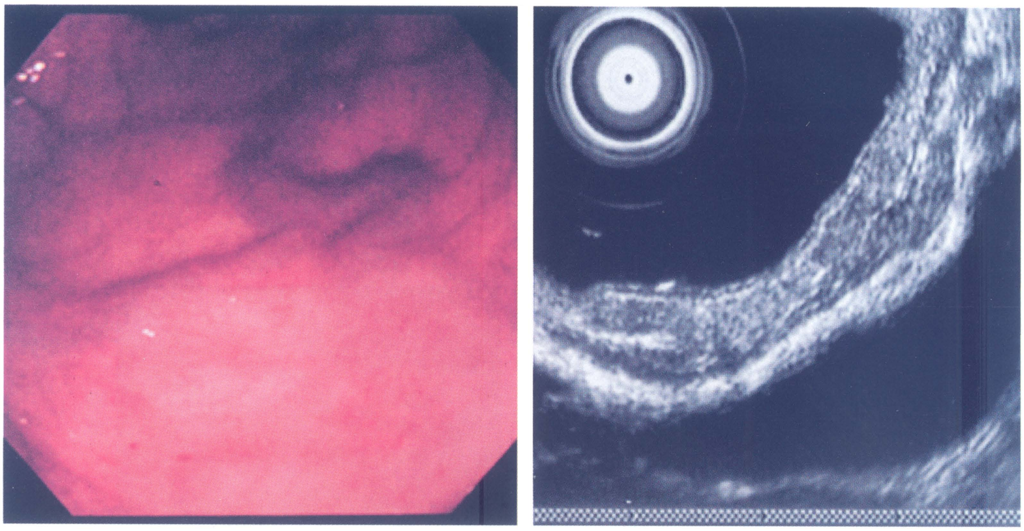

FIGURE 1 Endoscopic findings of rectal varices ($F_1V$).

the deaerated-water filling method in all patients while withdrawing the probe from the recto-sigmoid, observing mainly the SMVs. When the lumen was visualized in the longitudinal direction on US scan, its longest diameter was measured at right angles to the axial direction of the probe. Mainly superficial SMVs were evaluated, and perforating vessels and extramural perirectal veins were excluded.

The following four colonoscopic findings were obtained in the PH and control groups.

(1) $F_1$ varices ($F_1V$): Varices proximal to the anal border that are compatible with $F_1$ acceding to the Criteria for Evaluation of Endoscopic Findings of Varices [11] (Fig. 1).
(2) $F_2$ varices ($F_2V$): Varices similar to (1) that are compatible with $F_2$ or higher stage (Fig. 2).

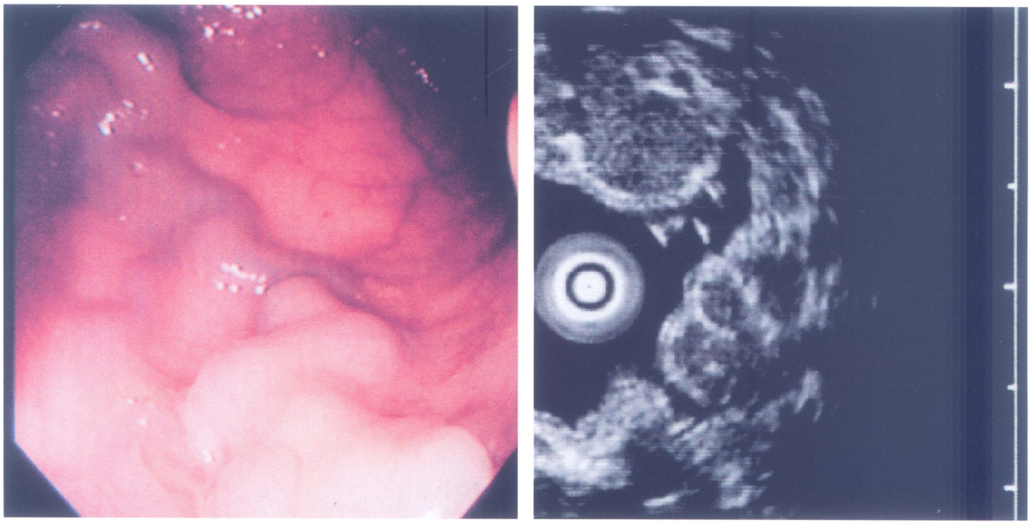

FIGURE 2 Endoscopic findings of rectal varices ($F_2V$).

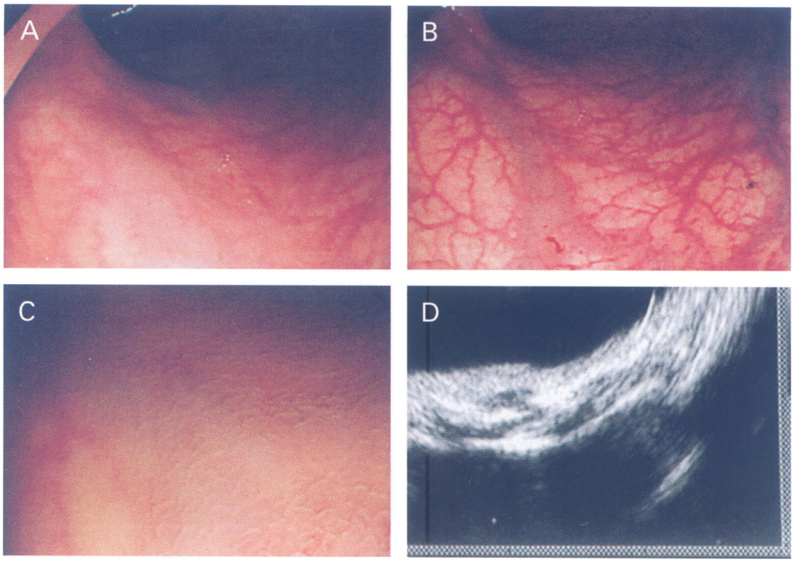

FIGURE 3 Endoscopic findings of blue vein.

(3) Blue vein (BV): Dilated and slightly elevated blue-tinted vein that comes into view after air insufflation but looks flattened (Fig. 3A–C).
(4) Angiectasia (AE): A cluster of spider angioma-like minute vessels.

On the other hand, the vessel visualized by EUS mainly in the submucosal layer was classified as SMV (Fig. 3D).

The spleen index (longest diameter by thickness of the spleen on the US scan: SI) was used as an evaluation variable for PH.

The study was broadly divided into two parts. In the first part, (1) the frequency of endoscopic changes and the rate of SMV visualization by EUS and (2) the SMV diameter determined by EUS were compared between the PH and the control groups. The RV were defined based on the SMV diameter. Next, (3) the relationship of the presence or absence of RV with clinical characteristics of patients in the PH group, (4) the relationship of the presence or absence of RV with hepatic and SI in the PH group, (5) the frequency of RV as classified by Child in patients with liver cirrhosis, (6) the frequency of RV by the severity of hepatic function impairment, and (7) the relationship of the presence or absence of RV with endoscopic findings in the PH group were investigated.

TABLE II Relationship of the frequency of endoscopic changes with the visualization rate of submucosal vein by EUS

| Endoscopic findings | Patients $N = 50$ (%) | Visualization rate of SMV (%) | Control $N = 25$ (%) | Visualization rate of SMV (%) |
|---|---|---|---|---|
| Varices | 12 (24) | 12 (24) | 0 | |
| $F_1V$ | 9 (18) | 9 (18) | 0 | |
| $F_2V$ | 3 (6) | 3 (6) | 0 | |
| Blue vein | 18 (36) | 18 (36) | 10 (40) | 10 (40) |
| Angiectasia | 3 (6) | 0 (0) | 0 | |
| Normal | 17 (34) | 14 (28) | 15 (60) | 7 (28) |
| Total | | 44 (88) | | 17 (68) |

SMV: submucosal vein.

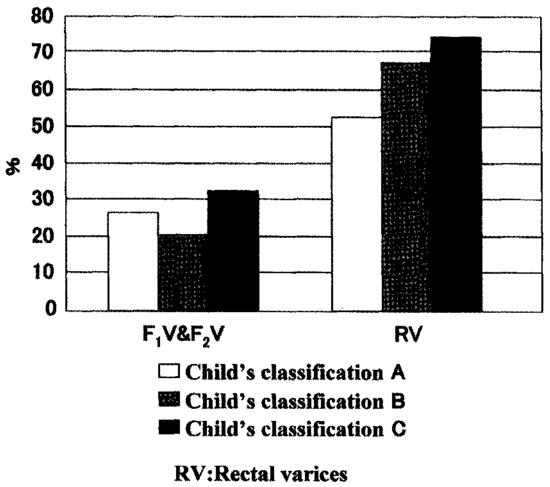

FIGURE 4 Incidence of $F_1V/F_2V$ and RV classified according to Child-Turcotte's classification of liver cirrhosis.

The significance of differences between the two groups was assessed by the $\chi^2$-test and Fisher's exact test, and the differences were considered significant when $p < 0.05$.

### RESULTS

(1) Relationship of the frequency of endoscopic changes with the visualization rate by EUS (Table II).

TABLE III Submucosal vein diameter

| Endoscopic findings | Patients $N = 44$ (mm) | Control $N = 17$ (mm) |
|---|---|---|
| $F_1V$ and $F_2V$ | 4.3 (3.2–7.5) | |
| Blue vein | 2.5 (1.2–3.5)* | 1.2 (0.8–1.5) |
| Invisible-SMV | 1.7 (0.7–2.8)* | 0.6 (0.4–0.8) |
| Total | 2.7 (0.7–7.5)* | 0.9 (0.4–1.5) |

SMV: submucosal vein; $*p < 0.01$.

$F_1V$ and $F_2V$ occurred in 12 patients (24%) in the PH group of whom 9 (18%) had $F_1V$ and 3 (6%) $F_2V$. Hemorrhage was observed in two of three $F_2V$ patients during the course of observation. Hemostasis was obtained in one of the three patients, but an ill general condition and unrest did not permit hemostatic treatment in one patient. As a result, the patient died. BV was seen in 18 patients (36%) and AE in 5 patients (10%), but none of them had hemorrhage. The rate of SMV visualization by EUS was 24% for (12) patients with $F_1V$ and $F_2V$ and 36% for all (18) patients with BV. SMV was visualized in a total of 44 patients (88%), including 14 patients (28%) in whom the endoscopic findings were not remarkable and SMV could be visualized by EUS alone (Fig. 4).

For the control group, on the other hand, the frequency of $F_1V$ and $F_2V$ was 0%, and the frequencies of BV and AE were 40% (10 patients) and 0%

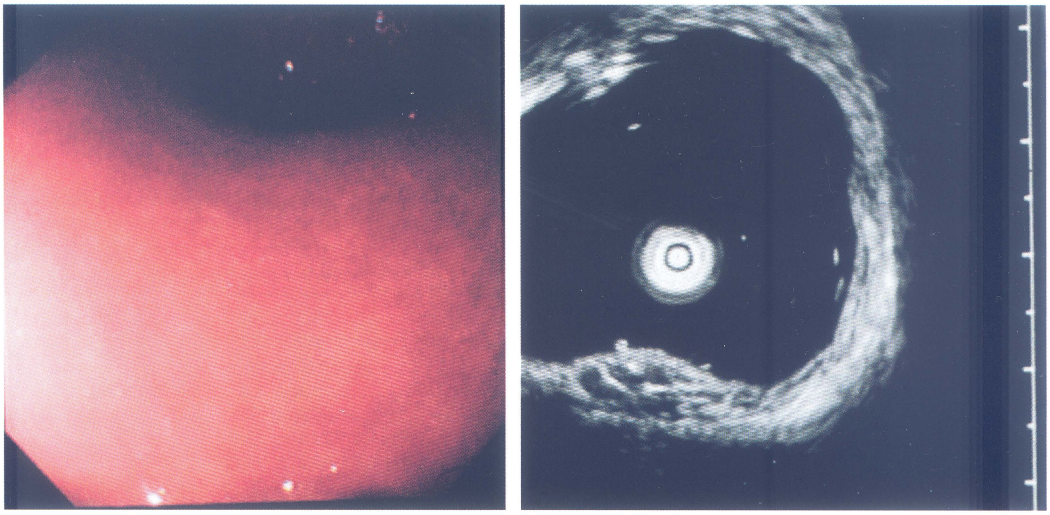

FIGURE 5 Endoscopic findings of dilated submucosal vein detected on EUS only.

(0 patient), respectively. The rate of SMV visualization by EUS was 40% (10 patients) for BV. SMV was visualized in a total of 17 patients (68%), including 7 patients (28%) in whom it was visualized by EUS alone. $F_1V$, $F_2V$, and AE were specific occurrences for the PH group, compared to the control group, but there was no significant difference between the PH and the control groups with respect to the frequency of BV. There was no significant difference again between the two groups in the rate of SMV visualization. The rate of SMV visualization was 28% higher in both groups for EUS than for endoscopy.

(2) SMV diameter (Table III).

The mean SMV diameter was 4.3 mm for $F_1V$ and $F_2V$, 2.5 mm for BV, and 1.7 mm for SMV visualized only by EUS, that is, invisible SMV (subsequently referred to as IV-SMV) in the PH group. The SMV diameters were significantly greater for the PH group, compared to 1.2 mm for BV and 0.6 mm for IV-SMV in the control group. All together, the mean SMV diameter was 2.7 mm (range 0.7–7.5 mm) for the PH group and 0.9 mm (range 0.4–1.5 mm), and it was again significantly greater for the PH group. In the control group, the longest SMV diameter was 1.5 mm, and no SMV greater than 2 mm in diameter was observed (Fig. 5). SMV 2 mm or more in diameter was defined as RV, and patients were divided into an RV(+) group of 31 patients (62%) and an RV(−) group of 19 patients (38%) to determine the relationship of RV with clinical characteristics of patients.

(3) Relationship of RV with demographic and clinical characteristics of patients (Table IV).

The RV(+) group had a mean age of 62.5 years, consisting of 21 males and 10 females, and the RV(−) group had a mean age of 67.9 years, consisting of 14 males and 5 females. There was no significant difference between the two groups with respect to the incidence of hepatopathy as the underlying disease, but the RV(+) group tended to include a larger number of patients with alcoholic liver cirrhosis (5/6). There was no significant difference between the RV(+) and the RV(−) groups with respect to the presence or absence of EV or hepatocellular carcinoma.

TABLE IV Relationship of rectal varices with demographic and clinical characteristics of the portal hypertensive group

| | RV(+) | RV(−) |
|---|---|---|
| Cases | 31 | 19 |
| Mean age | 62.5 (37–81) | 67.9 (46–79) |
| Sex: M/F | 21/10 | 14/5 |
| LC | 29 | 17 |
| HCV | 21 | 15 |
| HBV | 1 | 0 |
| Alcohol | 5 | 1 |
| NBNC | 2 | 1 |
| IPH | 2 | 1 |
| EHO | 0 | 1 |
| E. varices | 24 (77%) | 14 (74%) |
| Hepatoma | 17 (55%) | 10 (53%) |

RV (Rectal varices): submucosal vein exceeding 2 mm in diameter; LC: liver cirrhosis; IPH: idiopathic portal hypertension; EHO: extrahepatic portal vein obstruction; E: esophageal.

(4) Relationship of RV with hepatic reserve and SI in the PH group (Table V).

Serum albumin and cholinesterase were significantly lower for the RV(+) group than for the RV(−) group (3.2 vs 3.5 and 112 vs 165, respectively), and SI was significantly higher for the RV(+) group (74 vs 57). There was no significant difference between the two groups with respect to direct bilirubin, platelet count, prothrombin time, ICG or ammonia.

(5) Incidence of $F_1V/F_2V$ and RV classified according to Child-Turcotte's classification of liver cirrhosis (Fig. 4).

There was no difference in the incidence of $F_1V/F_2V$ with respect to type, while the incidence of RV which stood at 53% for A, 68% for B, and 75%

TABLE V Relationship of rectal varices with hepatic reserve and spleen index in the portal hypertensive group

| | RV(+) $N = 31$ | RV(−) $N = 19$ | $p$ value |
|---|---|---|---|
| Alb (g/dl) | 3.2 ± 0.4 | 3.5 ± 0.5 | < 0.05 |
| T-b (mg/dl) | 1.2 ± 0.7 | 1.2 ± 0.5 | NS |
| Ch-E (IU/l) | 112 ± 45 | 165 ± 64 | < 0.01 |
| Plt (× $10^4$) | 8.8 ± 3.0 | 10.3 ± 3.3 | NS |
| PT (%) | 69.9 ± 10 | 72.0 ± 12.0 | NS |
| $ICG_{R-15}$ (%) | 29.6 ± 13.9 | 26.2 ± 15.0 | NS |
| $NH_3$ (μg/dl) | 69.8 ± 31.0 | 72.3 ± 38.8 | NS |
| SI | 74 ± 20 | 57 ± 16 | < 0.01 |

RV: rectal varices; Alb: albumin; T-b: total bilirubin; Ch-E: cholinesterase; Plt: platelet; PT: prothrombin time; SI: spleen index.

TABLE VI Incidence of rectal varices by severity of liver impairment

| | | RV $N = 31$ (%) | $p$ |
|---|---|---|---|
| Alb (g/dl) | $>3.5$ | 8/17 (47.1) | NS |
| | $\leq 3.5$ | 23/33 (69.7) | |
| Plt ($\times 10^4$) | $\geq 10$ | 12/24 (50) | NS |
| | $<10$ | 19/26 (73.1) | |
| Ch-E (IU/l) | $\geq 140$ | 9/20 (45) | NS |
| | $<140$ | 22/30 (73.3) | |
| SI | $<60$ | 8/21 (38.1) | $<0.01$ |
| | $\geq 60$ | 23/29 (79.3) | |

RV: rectal varices; Alb: albumin; Plt: platelet; Ch-E: cholinesterase; SI: spleen index.

for C tended to increase with diminishing hepatic reserve.

(6) Relationship of the frequency of RV with the severity of hepatic function impairment (Table VI).

Assuming that the hepatic reserve is good if serum albumin, platelet count and serum cholinesterase are $>3.5$, $\geq 10^6$, and $\geq 140$, respectively, the frequency of RV tended to be higher for the group with poor hepatic reserve than for the group with good hepatic reserve. Assuming that PH is mild if SI is $<60$ and severe if SI is $\geq 60$, the frequency of RV is significantly higher for the group with severe PH than for the group with mild PH (79.3 vs 38.1%).

(7) Relationship of RV with endoscopic findings (Table VII).

Thirty-one patients were RV positive. $F_1V/F_2V$ was present in 12 patients (100%), and BV present in 14 of 18 patients (78%). Dilated SMV (Fig. 5) was observed with EUS in 5 of 17 patients (29%) with endoscopically normal mucosa. Hemorrhage was observed in two patients with $F_2V$. Varices in these patients gradually grew in size during observation.

TABLE VII Relationship of rectal varices with endoscopic findings

| | Endoscopic findings | | |
|---|---|---|---|
| | $F_1V$ and $F_2V$ $N = 12$ | Blue vein $N = 18$ | Normal $N = 17$ |
| RV(+) | 12 (2) | 14 | 5 |
| RV(−) | 0 | 4 | 12 |

RV: rectal varices; ( ): bleeding case.

One of them had SMV 5.9 mm in diameter, but hemorrhage could be stopped by EVL. The other patient had SMV 7.5 mm in diameter and could not be treated because of poor systemic condition.

### DISCUSSION

The colonoscopic findings of PH so far reported consist of (1) dendriform vasodilation, (2) spider angioma-like lesions, (3) dilated blue vein, and (4) varices. Dendriform vasodilation, a tree-shaped appearance of a dilated vessel seem through the mucosa. Dendriform vasodilation is the term used by Sai *et al.* [12]. Fujii *et al.* [13] describe it as a dilated fine branching vessel, and Motoyama *et al.* [1] call it merely a tree. It has been reported that the incidence of dendriform vasodilation is as high as 85–90% in patients with PH of cirrhosis, [1,12,13] but the diagnostic criteria are so vague that the supporting data lack objectivity. The spider angioma-like lesion is a red spot formed by a localized cluster of minute vessels. The spider angioma-like lesion is the term used by Sai *et al.* [12]. Fujii *et al.* and [13] Okawa *et al.* [14] describe it as vascular ectasia, and Sakai *et al.* [15] call it angiectasia (AE). It is said to be common in patients with PH of cirrhosis. The spider angioma-like lesion is specific for PH of cirrhosis, but its incidence so far reported widely varies from 6 to 63% [1,12–14]. It appears that the lesions are so small that they often escape detection. The dilated blue vein is a blue vein 2–3 mm in diameter. The blue vein is the term used by Sai *et al.* [12] and Fujii *et al.* [13]. They have reported the incidence is high, 30–51% in patients with PH and that it is highly specific for PH. It is liable to undergo morphological changes depending on the amount of air insufflation in the endoscopic examination. BV was detected in 36% of patients in the PH group and 40% in the control group. Any argument for its morphological invariability would seem hardly tenable, objectively. Varices are observed as a characteristic blue-tinted submucosal tumor-like elevation and, therefore, diagnosis is easy. The incidence of colo-rectal varices in PH patients reported overseas widely varies from 3.6 to 89% [6,16–21]. Its incidence is lower in Japan, at 16–21%, than in other countries [1,12,13]. It seems that varices are more specific and objective than other consequences of PH. As suggested by the fact that the four lesions are collectively called portal hypertensive colopathy, they are known to be associated with PH, but their mutual relationship and the process of progression still remain obscure in many respects. These lesions are thought to become aggravated with time as PH progresses, but the process of progression has not been elucidated in detail. A most clinically serious event that arises from these lesions is bleeding from ruptured varices, in particular, varices of the colon. Nakazawa *et al.* [22] described RV for the first time in 1982 in Japan. In later years, ruptured RV was reported [23,24]. One death occurred in our institution too. The incidence of hemorrhage so far reported is not high, 0.45–3.6% [25–27]. A copious hemorrhage may claim the patient occasionally [28]. It is important to observe the patient with a hemorrhage from RV as in the case of EV.

RV may be confused with internal hemorrhoids because it is located near the anus, but in actuality both the site and mechanism of development differ from RV to internal hemorrhoids. Hemorrhoids are not related with PH and are dilated existing hemorrhoidal veins that are localized in the submucosa of the anal canal. Hemorrhoids are a mere vascular cushion. RV, on the other hand, is the collateral that is formed between the superior rectal vein that develops superiorly from the canal in conditions of PH and returns to the internal iliac vein of the portal system and the inferior rectal vein that returns to the internal iliac vein of the inferior vena cava. This collateral circulation serves to decrease portal pressure [18,29]. RV arises from PH of liver cirrhosis in about 70% of patients [30]. Other causes of RV include postoperative intraperitoneal adhesion, mesenteric vein obstruction, congenital anomalies of the portal system, vascular deformation, and heart failure [30–34]. It is easy to visualize dilated, swollen vessels of the colon under endoscopic control as in the esophagus, but the incidence of RV in PH is about 20% in Japan [1,12,13].

Detecting dilated SMV before they progress to the extent that they can be visualized by endoscopy is very helpful in diagnosing RV early. Since the rectum is located near the anus, so that relatively easy access can be gained, examination of the rectal veins for morphological changes by the technique combining safe and non-invasive EUS is considered useful in pathological evaluation of PH.

Sai *et al.* [12] reported that the incidence of varices and BV combined was 46.7% for the PH group, compared to 0% for the control group. In the present study, it was a little higher at 60%. It should be noted, however, that BV was detected in 40% of patients in the control group unlike in the study by Sai *et al.* This discrepancy may in part be accounted for by the differing diagnostic criteria, but seems attributable in large part to the fact that the diagnosis of BV itself is not objective enough.

All $F_1V$, $F_2V$ and BV that were identified endoscopically in this study were visualized as SMV by EUS, but AE defied EUS. In this study, therefore, AE was not taken into account in the investigation by EUS.

Dhiman *et al.* [6,7] counted SMVs and determined their diameter and the rate of visualization using a dedicated EUS apparatus. According to them, SMVs were larger in number and size in the PH group than in the control group, and the rate of visualization was 75–85% for the PH group and 25–30% for the control group. The rate of visualization was high in both the PH and control groups. The reason seems that thinner SMVs (1 mm or less in diameter) could be visualized in our study because we used ultrasonic probes that provide an excellent scan under direct endoscopic control and reveal details of superficial lesions.

They state that SMVs more than 2 mm in diameter may be defined as RV both because SMVs more than 2 mm in diameter were not observed in the control group and because SMV was visualized in 25–30% of patients in the control group.

Naveau *et al.* [35,36] reported that RV was related with EVs 5 mm or more in maximum diameter or a history of hemorrhage from EV, but the present study indicated no close correlation between RV and a hemorrhage from EV or hepatocellular carcinoma. It should be noted, however, that SI was referred to as a parameter of portal tension in this study because it could not be measured accurately.

Considering that the frequency of RV was significantly higher for the group with severe splenoma with SI ≥ 60, it seems that RV is not so much affected by hepatic function as by the severity of PH.

Endoscopic injection sclerotherapy (EIS) was started by Wang *et al.* [37] in 1985, and endoscopic variceal ligation (EVL) was performed by Levine *et al.* [38] in 1993. Similar reports were published in Japan [9,10,18,19]. The efficacy of EIS and EVL involving limited invasion has been established gradually. Aethoxysklerol (AS), ethanolamine oleate with iopamidol (EOI), ethanol, and cyanoacrylate were used for sclerotherapy. Zenitani *et al.* [9] could not control hemorrhage in RV patients by extravascular injection of 1% AS, but succeeded in controlling it by intravascular injection of 5% EOI. They attribute failure in hemostasis to large calibers of vessels (5 mm for varices, 4 mm for perforated vessels) and consider pre- and postoperative EUS helpful in controlling hemorrhage. In light of our results, it seems advisable that appropriate therapy be chosen for RV as for EV after evaluation of affected blood vessels for size and condition by EUS. If affected vessels have a large caliber, combination therapy with EIS by intravascular injection seems useful. There were two patients with hemorrhage at our institution. RV was detected in about 30% of patients with endoscopically normal mucosa in the PH group. Since these patients can be considered to be at high risk of hemorrhage, EUS seems to be of great significance because it is helpful in the detection of RV. It is important that rectal EUS be performed aggressively in patients with PH accompanied by diminished hepatic reserve, EV, and splenomegaly and that SMV and surrounding vessels are evaluated periodically. Preventive EIS may be necessary for the treatment of $F_2$ or severer varices.

### CONCLUSIONS

EUS was helpful in staging PH. EUS is more useful than endoscopy in that it detects EV with the highest precision and provides for evaluation of PH patients at risk of hemorrhage.

### *Acknowledgements*

The authors want to express their deep appreciation to Prof. Yoshihiro Sakai at the Third Department of Internal Medicine, Toho University School of Medicine for his review of the manuscript. Appreciation is also due to Dr Sumio Fujinuma, Dr Kazuya Yoshimoto, Dr. Tadayoshi Kakemura, and our colleagues for their assistance in this investigation.

### *References*

[1] Motoyama, H., Ueki, J., Seki, K., *et al.*, (1998) "Portal hypertensive colopathy: A proposal of characteristic endoscopic findings", *Gastroenterol. Endosc.* **40**, 160–168.

[2] Yamakado, S., Kanazawa, H. and Kobayashi, M. (1995) "Portal hypertensive colopathy: endoscopic findings and the relation to portal pressure", *Intern. Med.* **34**, 153–157.

[3] Kozarek, R.A., Botoman, V.A., Bredfeldt, J.E., *et al.*, (1991) "Portal hypertensive colopathy: prospective study of colonoscopy in patients with portal hypertension", *Gastroenterology* **101**, 1192–1197.

[4] Massachusette General Hospital (1954) "Case records of the Massachusetts general hospital. Case 40102", *N. Engl. J. Med.* **250**, 434–437.

[5] Hochi, Y., Watanabe, S. and Sakai, Y. (1992) "Urgent endoscopy for the lower gut bleeding", *Jpn J. Acute Med.* **16**, 21–26.

[6] Dhiman, R.K., Choudhuri, G., Saraswat, V.A., *et al.*, (1993) "Endoscopic urtrasonographic evaluation of the rectum in cirrhotic portal hypertension", *Gastrointest. Endosc.* **34**, 546–549.

[7] Dhiman, R.K., Saraswat, V.A., Choudhuri, G., *et al.*, (1999) "Endosonographic, endoscopic, and histologic evaluation of alterations in the rectal venous system in patients with portal hypertension", *Gastrointest. Endosc.* **49**, 218–227.

[8] Malde, H., Nagral, A., Shah, P., *et al.*, (1993) "Detection of rectal and pararectal varices in patients with portal hypertension: Efficacy of transvaginal sonography", *AJR* **161**, 335–337.

[9] Zeniya, A., Odashima, M., Takahashi, H., *et al.*, (1997) "A case of rectal varices treated with endoscopic injection sclerotherapy", *Jpn J. Gastroenterol.* **94**, 38–43.

[10] Shudo, R., Yazaki, Y., Sakurai, S., *et al.*, (2001) "Combined endoscopic variceal ligation and sclerotherapy for bleeding rectal varices associated with primary billiary cirrhosis: a case showing a long-lasting favorable response", *Gastrointest. Endosc.* **53**, 661–665.

[11] Japanese Research Society for Portal Hypertension (1980) "The general rules for recording endoscopic findings on esophageal varices", *Jpn J. Surg.* **10**, 84–87.

[12] Hung-Fei, Tsai (1991) "Endoscopic study of vascular lesions of colon in portal hypertension", *Jpn J. Gastroenterol. Surg.* **24**, 1242–1250.

[13] Fujii, T., Maruoka, M., Takeuchi, Y., *et al.*, (1994) "Endoscopic study of vascular lesions of the colonic mucosa in patients with portal hypertension", *Gastroenterol. Endosc.* **36**, 337–342.

[14] Okawa, K., Aoki, T., Ikeda, Y., *et al.*, (1992) "Prospective study of colonic vascular ectasia in patients with liver cirrhosis", *Gastroenterol. Endosc.* **34**, 48–56.

[15] Sakai, Y., Suda, H., Kobayashi, H., *et al.*, (2000) "Angiectasia of the colon and rectum", *Stomach Intest.* **35**, 763–769.

[16] Rabinovits, M., Schade, R.R., Dindzans, V.J., *et al.*, (1990) "Colonic disease in cirrhosis. An endoscopic evaluation in 412 patients", *Gastroenterology* **99**, 195–199.

[17] Kinkhabwala, M., Mousavi, A., Iyer, S., *et al.*, (1977) "Bleeding ileal varicosity demonstrated by transhepatic portography", *AJR* **129**, 514–516.

[18] Hosking, S.W., Smart, H.L., Johnson, A.G., *et al.*, (1989) "Anorectal varices, haemorrhoids, and portal hypertension", *Lancet* **18**, 349–352.

[19] Chawla, Y.K. and Dilawari, J.B. (1991) "Anorectal varices: their frequency in cirrhotic and non-cirrhotic portal hypertension", *Gut* **32**, 309–311.

[20] Wang, T.F., Lee, F.Y., Tsai, Y.T., *et al.*, (1992) "Relationship of portal pressure, anorectal varices and hemorrhoids in cirrhotic patients", *J. Hepatol.* **15**, 170–173.

[21] Goenka, K., Kochhar, R., Nagi, B., *et al.*, (1991) "Rectosigmoid varices and other mucosal changes in patients with portal hypertension", *Am. J. Gastroenterol.* **86**, 1185–1189.

[22] Nakazawa, S., Oida, T., Onizuka, T., *et al.*, (1982) "Rectosigmoid colonic varices, report of a case", *Stomach Intest.* **17**, 97–102.

[23] Kojima, T., Onda, M., Tajiri, T., *et al.*, (1996) "A case of massive bleeding from rectal varices treated with endoscopic variceal ligation (EVL)", *Jpn J. Gastroenterol.* **93**, 114–119.

[24] Fujikawa, T., Ohmasa, R., Masuda, K., *et al.*, (1994) "Two cases of bleeding rectal varices developed during follow up of sclerotherapy for esophageal varices", *Gastroenterol. Endosc.* **36**, 51–57.

[25] McCormack, T.T., Bailey, H.R., Simms, J.M., *et al.*, (1984) "Rectal varices are not piles", *Br. J. Surg.* **71**, 163.

[26] Johansen, K., Bardin, J. and Orloff, M.J. (1980) "Massive bleeding from hemorrhoidal varices in portal hypertension", *JAMA* **244**, 2084–2085.

[27] Wilson, S.E., Stone, R.T., Christie, J.P., *et al.*, (1979) "Massive lower gastrointestinal bleeding from intestinal varices", *Arch. Surg.* **114**, 1158–1161.

[28] Watanabe, K., Kida, Y., Okudaira, M., *et al.*, (1992) "Autopsy links massive anorectal bleeding with sclerotherapy for gastroesophageal varices", *Kitasato Med.* **22**, 196–201.

[29] Weiserbs, D.B., Zfass, A.M. and Messmer, J. (1986) "Control of massive hemorrhage from rectal varices with sclerotherapy", *Gastrointest. Endosc.* **32**, 419–421.

[30] Takano, H., Yoshikawa, T., Takahashi, S., *et al.*, (1989) "Rectosigmoid colonic varices: an often unrecognized cause of lower gastrointestinal bleeding", *Jpn J. Gastroenterol.* **80**, 1310–1315.

[31] Moncure, A.C., Waltman, A.C., Vandersalm, T.J., *et al.*, (1976) "Gastrointestinal hemorrhage from adhesion-related mesenteric varices", *Ann. Surg.* **183**, 24–29.

[32] Vella-Camilleri, F.C., Friedrich, R. and Vento, A.O. (1986) "Diffuse colonic varices: an uncommon case of intestinal bleeding", *Am. J. Gastroenterol.* **81**, 492–494.

[33] Arbona, J.L., Lichtenstein, J.E., Spies, J.B., *et al.*, (1987) "Colonic varices as a complication of colonic surgery", *Gastrointest. Radiol.* **12**, 350–352.

[34] Weingart, J., Hochter, W. and Ottenjann, R. (1982) "Varices of the entire colon—an unusal cause of recurrent intestinal bleeding", *Endoscopy* **14**, 60–70.

[35] Naveau, S., Bedossa, P., Poynard, T., *et al.*, (1991) "Portal hypertensive colopathy. A new entry", *Dig. Dis. Sci.* **36**, 1774–1781.

[36] Naveau, S., Poynard, T., Pauphilet, C., *et al.*, (1989) "Rectal and colonic varices in cirrhosis", *Lancet* **1**, 624.

[37] Wang, M., Desigan, G., Dunn, D., *et al.*, (1985) "Endoscopic sclerotherapy for bleeding rectal varices: A case report", *Am. J. Gastroenterol.* **80**, 779–780.

[38] Levine, J., Tahiri, A. and Banerjee, B. (1993) "Endoscopic ligation of bleeding rectal varices", *Gastrointest. Endosc.* **39**, 188–190.

